# Real-world evaluation of bevacizumab plus platinum and paclitaxel for advanced cervical cancer: a single-arm study from Syria

**DOI:** 10.3332/ecancer.2026.2066

**Published:** 2026-01-22

**Authors:** Bana Mohamad, Maher Saifo

**Affiliations:** 1 Department of Oncology, Damascus University, Al Qabo, Main Square, Homs, Syria; 2 Department of Oncology, Damascus University, Harasta Medical Staff Residence, Al Beiruni Hospital, Damascus, Syria

**Keywords:** cervical cancer, bevacizumab, platinum chemotherapy, RMST, real-world data

## Abstract

**Background:**

Bevacizumab combined with chemotherapy has become a standard first-line treatment for advanced cervical cancer following the Gynecologic Oncology Group (GOG)-240 trial. However, real-world data from low-resource settings, such as Syria, remain limited.

**Objective:**

To evaluate the clinical effectiveness of bevacizumab with paclitaxel and either cisplatin or carboplatin in Syrian patients with metastatic or recurrent cervical cancer, using both median progression-free survival (PFS) and restricted mean survival time (RMST) analysis.

**Methods:**

A single-arm prospective/retrospective study was conducted at Al-Beiruni University Hospital between January 2023 and December 2024, including 64 patients. Treatment was administered every 21 days with a median of eight cycles per patient (range: 4–20). PFS was estimated using Kaplan–Meier analysis, while RMST was calculated at 3, 6, 9 and 12 months using MedCalc and Python-based tools. Results were compared to the GOG-240 trial as a historical reference.

**Results:**

The median PFS was 10.1 months (95% CI: 9.46–10.75), representing a 23.2% increase over GOG-240. RMST analysis demonstrated a consistent survival benefit, with a 25.7% gain at 12 months. The objective response rate reached 61.3%, compared to 48% in the reference trial.

**Conclusion:**

This study supports the effectiveness of bevacizumab-based therapy as a first-line option in the Syrian setting, showing sustained clinical benefit and reinforcing the relevance of international guidelines in local oncology practice.

## Background

Cervical cancer is one of the most common malignancies affecting women globally, especially in low- and middle-income countries (LMICs) [1, 2]. Persistent infection with high-risk types of human papillomavirus (HPV), particularly types 16 and 18, is the primary etiological factor [3]. Other risk factors include smoking [4], long-term use of oral contraceptives [5], early sexual activity and immunosuppression [6]. Despite significant advances in prevention through HPV vaccination and screening programs, many women are still diagnosed at advanced stages, where treatment options are limited and prognosis is poor [7].

Standard treatment for advanced, metastatic or recurrent cervical cancer has historically included chemotherapy, with platinum-based agents forming the backbone. The incorporation of anti-angiogenic therapy, particularly bevacizumab, has represented a significant advancement in recent years. Bevacizumab is a monoclonal antibody targeting vascular endothelial growth factor-alpha, a key mediator of angiogenesis. The landmark Gynecologic Oncology Group (GOG)-240 trial demonstrated that adding bevacizumab to chemotherapy in the first-line recurrent/metastatic setting significantly improves overall and progression-free survival (PFS) [8].

More recently, immunotherapy with checkpoint inhibitors, in combination with chemotherapy with or without bevacizumab, has become part of the current standard of care for recurrent or metastatic cervical cancer in many high-income countries (HICs) [9]. However, access to immunotherapy remains very limited in LMICs, where barriers such as cost, infrastructure and drug availability continue to restrict implementation [10].

This study aims to evaluate the clinical outcomes of bevacizumab-based combination therapy in the context of a Syrian tertiary care centre and to compare them with global reference data.

## Methods

This is a prospective/retrospective study conducted at Al-Beiruni University Hospital, a major tertiary referral oncology centre in Syria. The study aimed to evaluate the real-world effectiveness of first-line therapy combining bevacizumab with platinum and paclitaxel in patients with metastatic or recurrent cervical cancer.

All patients received first-line systemic therapy consisting of bevacizumab in combination with paclitaxel and a platinum-based agent—either cisplatin or carboplatin—administered in 21-day cycles. Bevacizumab was typically given at a dose of 15 mg/kg intravenously every 3 weeks, while paclitaxel was administered at 175 mg/m² over 3 hours. The choice between cisplatin and carboplatin was made based on renal function, toxicity profile and physician discretion. Dose modifications and delays were implemented when required, in accordance with toxicity grading and patient condition. In the prospective cohort, drug administration and adverse events were recorded in real time. For retrospective cases, detailed chart reviews were conducted to verify adherence to the treatment regimen.

A total of 78 patient charts were reviewed. Fourteen patients were excluded for the following reasons: absence of histological confirmation of metastatic or recurrent disease (*n* = 4), non-metastatic or non-recurrent cervical cancer (*n* = 3), presence of active bleeding or non-healed wounds (*n* = 2), prior systemic therapy different from platinum–paclitaxel plus bevacizumab (*n* = 3) and Eastern Cooperative Oncology Group (ECOG) performance status >2 (*n* = 2). The final study population, therefore, consisted of 64 patients.

**Figure 1.**
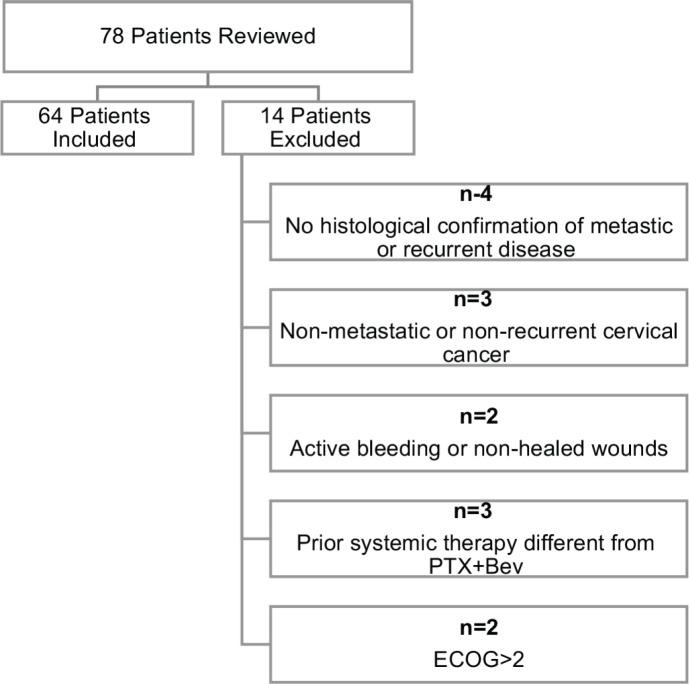
Flow diagram of patient inclusion and exclusion.

To reduce the risk of bias, data collection was standardised using predefined case report forms. In the retrospective cohort, double chart reviews were performed by two independent investigators to ensure data accuracy and consistency. In the prospective cohort, treatment delivery and adverse events were recorded in real time. Patient eligibility was strictly defined by the inclusion/exclusion criteria and treatment protocols followed institutional standards, minimising variability in clinical management. Furthermore, once the predefined eligibility criteria had been applied prior to study initiation, all 64 patients were analysed according to the intention-to-treat (ITT) principle. No patients were excluded after study commencement and no selective omission based on clinical course or treatment response was undertaken. The study flow is illustrated in Figure 1.

### Study design and period

The study followed a prospective single-arm design, with an additional retrospective extension aimed at enhancing the statistical power and precision of survival estimates by increasing the proportion of analysable progression events and reducing the impact of censoring. The timeframe covered cases treated between January 2023 and December 2024.

### Inclusion and exclusion criteria

Patients were eligible for inclusion if they had a histologically confirmed diagnosis of cervical cancer and presented with either metastatic or recurrent disease. All included patients had received first-line systemic therapy consisting of bevacizumab in combination with paclitaxel and a platinum-based agent. Additionally, eligibility required the availability of complete follow-up records or sufficient data to assess treatment response, defined as having documented imaging results (CT, MRI or PET), clinical assessments and laboratory tests at baseline and at least one follow-up time point after therapy initiation.

Patients were excluded if they presented with active bleeding, had non-healing wounds, had received any prior systemic first-line therapy for metastatic or recurrent cervical cancer (other than the study regimen) or had an ECOG > 2.

### Data collection and variables

Among the included patients, 24 were followed prospectively from January 2024 to December 2024, while retrospective data were collected for 40 patients from medical records at Al-Beiruni University Hospital covering the period from January 2023 until the end of the study in December 2024.

Data collected included patient demographics, tumour characteristics, prior treatments, comorbidities, laboratory values, imaging results and clinical outcomes. Standardised case report forms were used to ensure consistency across prospective and retrospective cohorts.

### Statistical analysis

Kaplan–Meier analysis was used to estimate PFS, and 95% confidence intervals were calculated. Restricted mean survival time (RMST) was computed at multiple predefined time points (3, 6, 9 and 12 months) to complement and validate median-based findings. All statistical analyses were conducted using MedCalc Statistical Software (version 23.2.1) and custom Python scripts (Python 3.10) for RMST area-under-the-curve (AUC) computations and curve digitisation. The assumption of proportional hazards was not formally tested in this study. As such, the RMST method was chosen specifically to provide a robust and assumption-free alternative for time-to-event comparisons.

## Results

A total of 64 patients with metastatic or recurrent cervical cancer were included in the final analysis. All patients received first-line systemic therapy with a combination of bevacizumab, paclitaxel and either cisplatin or carboplatin. The analysis was conducted based on the ITT principle, meaning that all eligible patients who initiated treatment were included in the analysis regardless of treatment completion, adherence or follow-up status.

The objective response rate (ORR) reached 61.3%, assessed according to Response Evaluation Criteria in Solid Tumours version 1.1, consistent with the methodology used in the GOG-240 trial. Tumour response was evaluated using imaging studies (CT or MRI) at baseline and every three months of therapy. For comparison, the ORR in the GOG-240 reference trial was 48%, highlighting the higher response observed in our cohort.

Patients received a median of 8 treatment cycles (range: 4–20), depending on clinical status, treatment response and tolerability.

### Patient characteristics

The study cohort included 64 patients, with demographic and clinical characteristics summarised in Table 1. The majority had recurrent disease (59.4%), while 40.6% presented with *de novo* metastatic disease. A significant proportion were smokers with a performance status of 0–1. Among metastatic cases, lung involvement was the most common site. At the time of analysis, 20.3% of patients were censored, while 79.7% had experienced disease progression. To address the limitations imposed by scheduled imaging intervals (every 3 months), a manual time-alignment method was applied to better approximate progression dates and reduce artificial drops in Kaplan–Meier curves, ensuring a more accurate survival analysis.

### Progression-free survival

**Figure 2.**
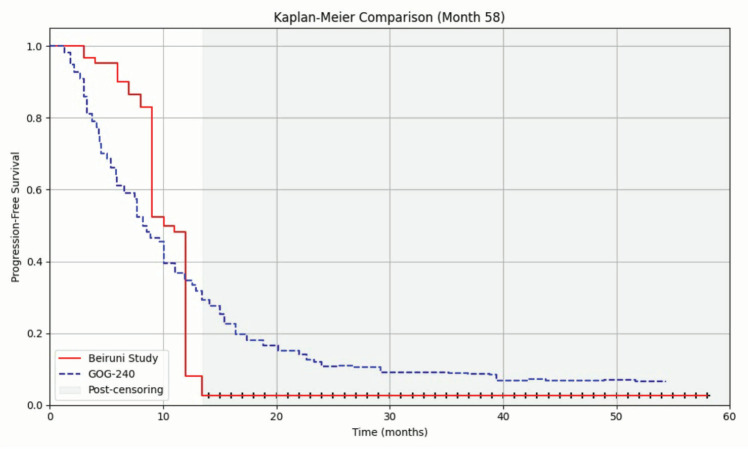
Kaplan–Meier curve of the Al-Beiruni University Hospital cohort and the digitised Kaplan–Meier curve from the GOG-240 trial. Post-censoring begins after the last observed event at month 14.

**Figure 3.**
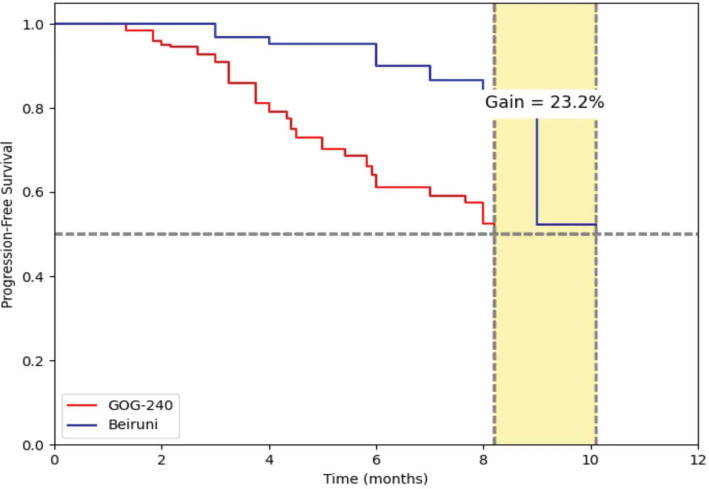
Median PFS in the Al-Beiruni cohort showed a 23.2% gain (1.9 months) compared to the GOG-240 curve. RMST analysis is required to confirm the reliability of this observed advantage.

The median PFS, estimated using the Kaplan–Meier method, was 10.101 months (95% CI: 9.457–10.745), as shown in Figure 2. Compared to the reference value reported in the GOG-240 trial (8.2 months), the clinical setting at Al-Beiruni Hospital demonstrated a gain of approximately 23.2%, corresponding to an absolute improvement of 1.9 months in median PFS (Figure 3). Considering the potential biases introduced by infrequent assessment intervals (typically every three months), a RMST analysis was performed to validate that the observed gain exceeded the expected margin of error. Treatment discontinuation was predominantly due to radiologically confirmed disease progression. A smaller proportion of patients discontinued therapy because of treatment-related toxicities or based on patient/physician decision. Due to the retrospective nature of the study, detailed documentation of discontinuation causes was not uniformly available across all patient records.

### RMST gain analysis

**Figure 4.**
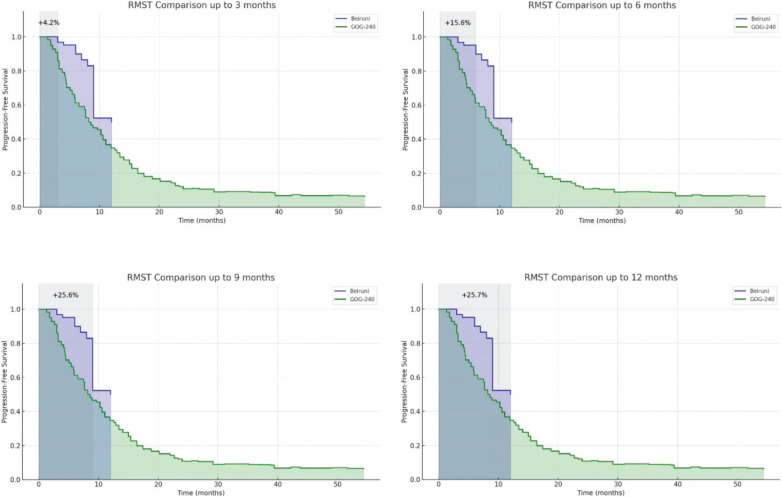
Area under the Kaplan–Meier curves for progression-free survival (PFS) in the Al-Beiruni cohort versus the digitised GOG-240 trial arm. RMST gain over multiple time points (e.g., 3, 6, 9 and 12 months) shows a consistently increasing survival benefit in the Al-Beiruni cohort, peaking at a 25.7% advantage at 12 months.

The RMST analysis was conducted by precisely calculating the AUC for both the Kaplan–Meier curve of the current study and the digitised Kaplan–Meier curve from the GOG-240 reference trial. As shown in Figure 4, the results demonstrate a cumulative and increasing survival gain, persisting even beyond the estimated median PFS. At 12 months, the RMST gain reached 25.1%, reinforcing the superiority of the observed outcomes beyond potential trial-related biases and confirming the effectiveness of the treatment regimen in the Syrian clinical setting, in line with the findings reported in the reference study. A dynamic simulation video was also generated to illustrate the behaviour of the Kaplan–Meier curves from both cohorts over a synchronised and clinically meaningful time scale.

## Adverse events

The Adverse events were monitored as part of routine clinical follow-up and documented based on available clinical records. The most frequently observed toxicity was neutropenia, occurring in 25 patients (40%), consistent with the known hematologic effects of platinum-based chemotherapy. Hypertension was the second most common adverse effect, reported in 18 patients (28%), followed by bleeding events in five patients (8%). Less frequent but clinically significant events included the occurrence of thromboembolic complications in three patients (5%) and fistula formation in two patients (3%), both of which are recognised risks associated with bevacizumab therapy. No unexpected toxicities were observed.

These findings are illustrated in Table 2, which summarises the distribution of adverse effects across the study population.

## Discussion

The findings of this study demonstrate a favourable PFS outcome among patients receiving bevacizumab in combination with platinum and paclitaxel at Al-Beiruni University Hospital. The observed median PFS of 10.1 months exceeded that of the historical control arm in the GOG-240 trial (8.2 months), suggesting potential real-world effectiveness in the Syrian clinical setting.

However, direct statistical comparison between our cohort and the GOG-240 trial is inherently limited by the absence of individual patient data (IPD), differences in baseline demographics and variability in healthcare infrastructure. Despite these encouraging findings, several limitations should be acknowledged. The retrospective nature of most of the cohort introduces an inherent risk of selection and information bias. The relatively small sample size (*n* = 64) further restricts the generalisability of the findings and the short follow-up period may have prevented the capture of long-term outcomes or late adverse events. Taken together, these methodological limitations necessitate cautious interpretation of the results and highlight the importance of validating our findings in larger, prospective studies.

Moreover, while median PFS is widely used as a summary endpoint in oncology trials, it has recognised limitations—particularly in non-proportional hazard scenarios or when survival curves diverge after the median point. Such limitations may obscure important long-term effects of treatment.

To address these concerns, we employed the RMST as a complementary analytic method. RMST provides an interpretable and statistically robust metric that quantifies the average event-free time within a pre-specified time window. Unlike median-based measures, RMST accounts for the entire shape of the survival curve, offering a more comprehensive view of therapeutic benefit. Our analysis revealed a consistent and growing RMST advantage in the local cohort compared to the GOG-240 reference arm, particularly within the first 12 months of follow-up. This methodological approach aligns with contemporary recommendations for non-randomised or single-arm studies, where direct head-to-head comparisons may not be feasible. It also provides a rational framework for contextualising international evidence within local healthcare environments that may differ in terms of diagnostic capacity, patient access and treatment continuity.

Furthermore, the observed improvement in both PFS and ORR in our cohort may reflect regional variations in patient selection, tumour biology or supportive care infrastructure. The RMST-based evaluation reinforces the clinical benefit of the regimen and mitigates biases that could arise from using median-based endpoints alone. Nonetheless, the single-arm design and lack of IPD from the GOG-240 trial limit the ability to draw definitive comparative conclusions.

In addition to survival benefits, the safety profile of the treatment regimen was consistent with previously reported data. Neutropenia and hypertension were the most commonly observed adverse effects, occurring in 40% and 28% of patients, respectively. Although thromboembolic events and fistula formation were less frequent (5% and 3%, respectively), they remain clinically significant risks associated with bevacizumab. Importantly, no unexpected toxicities were encountered and all adverse effects were managed according to institutional protocols. These findings reinforce the feasibility of implementing this regimen in resource-limited settings, with manageable safety outcomes.

In conclusion, these findings strengthen the rationale for using bevacizumab-based combination therapy in advanced cervical cancer in resource-limited settings and highlight the value of integrating RMST analysis into future oncology studies, especially where randomised controlled trials are not practical.

## Conclusion

This study supports the real-world clinical effectiveness of bevacizumab combined with platinum and paclitaxel as a first-line treatment in patients with metastatic or recurrent cervical cancer treated at Al-Beiruni University Hospital. The progression-free survival benefit, supported by both median and RMST-based analyses, reinforces the validity of adopting this regimen in resource-constrained settings. These findings provide additional evidence supporting the broader applicability of international trial results to local clinical environments and emphasise the importance of integrating advanced survival metrics in future comparative studies.

## Limitations of the study

While this study provides valuable insights into the real-world effectiveness of bevacizumab-based therapy in a Syrian clinical setting, several limitations should be noted. The single-center design may limit the generalisability of the findings to broader populations or healthcare environments. Additionally, the absence of a concurrent control arm necessitated comparisons with historical trial data, which may not fully account for differences in patient characteristics or clinical management. Resource constraints, including limited access to bevacizumab due to economic challenges and sanctions, affected treatment continuity for some patients and contributed to a modest sample size.

Nevertheless, the integration of both prospective and retrospective data helped mitigate this limitation and enhance statistical reliability. Finally, while Kaplan–Meier and RMST analyses were used to evaluate survival outcomes, proportional hazards assumptions were not formally assessed. However, RMST provided a robust and assumption-free alternative to support the validity of our conclusions.

## Conflicts of interest

The authors declare that they have no competing interests.

## Funding

None.

## Ethics approval and consent to participate

Informed consent was obtained from all patients who were followed prospectively for one year. In addition, formal approval was granted by Al-Beiruni University Hospital to access patient records retrospectively. All procedures were conducted in full compliance with ethical and legal guidelines, with strict adherence to patient confidentiality and data protection standards.

## Availability of data and materials

The data sets used and/or analysed during the current study are available from the corresponding author on a reasonable request.

## Trial registration

This study was not prospectively registered in an international clinical trial registry. Ethical approval was obtained from the Faculty of Medicine, Damascus University (Decision No. 243, dated November 1, 2023), and was subsequently approved by the Council for Scientific Research and Postgraduate Studies (Decision No. 1022, dated December 18, 2023), and by the University Presidency (Decision No. 64, dated January 15, 2024). The study was approved by the Institutional Review Board at Al-Beiruni University Hospital. All procedures conformed to the ethical standards of the Helsinki Declaration.

## Figures and Tables

**Table 1.** Patients characteristics.

Characteristic		Value/Distribution
Number of patients		64
Age (median, range)		56 years (47–70)
Smoking status	Smokers	47 (73.4%)
	Non-smokers	17 (26.6%)
Performance status	0	17 (26.5%)
	1	39 (61.0%)
	2	8 (12.5%)
Disease status	Recurrent cervical cancer	38 (59.38%)
	*De novo* metastatic cervical cancer	26 (40.63%)
Metastatic sites (*n* = 26)	Liver metastases	4 (15.4%)
	Bone metastases	6 (23.1%)
	Pulmonary metastases	16 (61.5%)
Histology (*n* = 55)	Squamous cell carcinoma	36 (65.5%)
	Adenocarcinoma	15 (27.5%)
	Other	4 (7%)
Survival status at analysis	Censored (under observation)	13 (20.31%)
	Event (disease progression)	51 (79.69%)

**Table 2.** Adverse events.

Adverse effect	Number of patients	Percentage
Neutropenia	25	40%
Hypertension	18	28%
Bleeding	5	8%
Thromboembolic events	3	5%
Fistula formation	2	3%
